# Supplementary material for: Mechanisms governing the pioneering and redistribution capabilities of the non-classical pioneer PU.1
Source: Nat Commun. 2020 Jan 21;11:402. doi: 10.1038/s41467-019-13960-2 (PMC6972792; doi:10.1038/s41467-019-13960-2)
Supplement: Supplementary file 7 — Source data [file 41467_2019_13960_MOESM7_ESM.zip › Source_Data/Figure6/Figure6B_MotifScanOutput/ETS1_PU.1/homerResults/motif22.similar.html]

motif22

## Information for motif22

G
T
A
C
T
C
G
A
T
C
G
A
G
A
T
C
G
T
C
A
C
T
A
G
A
G
T
C
G
T
C
A
C
T
A
G
  
Reverse Opposite:  

A
G
T
C
C
A
G
T
C
T
A
G
A
G
T
C
A
C
G
T
C
A
T
G
A
G
C
T
A
G
C
T
C
A
T
G
  

|  |  |
| --- | --- |
| p-value: | 1e-105 |
| log p-value: | -2.419e+02 |
| Information Content per bp: | 1.633 |
| Number of Target Sequences with motif | 5791.0 |
| Percentage of Target Sequences with motif | 36.58% |
| Number of Background Sequences with motif | 8589.4 |
| Percentage of Background Sequences with motif | 26.78% |
| Average Position of motif in Targets | 99.9 +/- 56.4bp |
| Average Position of motif in Background | 100.3 +/- 65.9bp |
| Strand Bias (log2 ratio + to - strand density) | -0.0 |
| Multiplicity (# of sites on avg that occur together) | 1.23 |
| Motif File: | file (matrix) reverse opposite |

### Similar de novo motifs found

|  |  |  |  |  |  |  |  |
| --- | --- | --- | --- | --- | --- | --- | --- |
| Rank | Match Score | Redundant Motif | P-value | log P-value | % of Targets | % of Background | Motif file |
| 1 | 0.770 | C T G A C T G A A G T C T C G A A C T G G A T C G A C T C T A G | 1e-55 | -126.742986 | 21.65% | 15.75% | motif file (matrix) |
| 2 | 0.852 | G A T C C T G A T G C A A G T C C T G A A C T G A G T C | 1e-37 | -86.932027 | 29.49% | 23.96% | motif file (matrix) |
